# Dermatitis as a component of the fetal inflammatory response syndrome is associated with activation of Toll-like receptors in epidermal keratinocytes

**DOI:** 10.1111/j.1365-2559.2006.02542.x

**Published:** 2006-11

**Authors:** Y M Kim, R Romero, T Chaiworapongsa, J Espinoza, G Mor, C J Kim

**Affiliations:** 1 Perinatology Research Branch, National Institute of Child Health and Human Development, National Institutes of Health, Department of Health and Human Services Bethesda, MD and Detroit, MI; 2 Department of Pathology, Wayne State University School of Medicine Detroit, MI; 3 Center for Molecular Medicine and Genetics, Wayne State University School of Medicine Detroit, MI; 4 Department of Obstetrics and Gynecology, Wayne State University School of Medicine Detroit, MI; 5 Department of Obstetrics and Gynecology, Yale University School of Medicine New Haven, CT, USA

**Keywords:** chorioamnionitis, dermatitis, fetal dermatitis, fetal inflammatory response syndrome, fetal skin, keratinocytes, Toll-like receptors

## Abstract

**Aims:**

Microbial invasion of the amniotic cavity (MIAC) elicits a fetal inflammatory response such as funisitis and chorionic vasculitis. However, little is known about the changes of fetal skin during MIAC. Toll-like receptors recognize microbial products and initiate an immune response. The aims of this study were to examine histopathological features of fetal skin exposed to MIAC and to assess the changes in Toll-like receptor (TLR)-2 and TLR-4 expression.

**Methods and results:**

Skin samples were obtained from fetal autopsies (*n* = 12). The cases were classified according to the presence (*n* = 8) or absence (*n* = 4) of acute chorioamnionitis and analysed by immunohistochemistry using a panel of antibodies. Leucocytic infiltrates into the superficial dermis were observed in cases with chorioamnionitis; the majority of inflammatory cells were neutrophils, lymphocytes and histiocytes. TLR-2 immunoreactivity in the skin was stronger in fetuses with chorioamnionitis than in those without this condition. However, immunoreactivity of TLR-4 in the fetal skin was constitutively expressed, regardless of the presence or absence of chorioamnionitis.

**Conclusions:**

This study demonstrates for the first time that fetal dermatitis can be detected and is part of the fetal inflammatory response syndrome (FIRS). We propose that this ‘FIRS-associated fetal dermatitis’ is a fetal counterpart of chorioamnionitis.

## Introduction

Microbial invasion of the amniotic cavity (MIAC) generates a unique environment for the propagation of infection and inflammation in both the mother and fetus. Fetal and maternal inflammatory responses are closely linked to preterm labour and delivery and have been associated with increased perinatal morbidity (neonatal sepsis, congenital pneumonia, necrotizing enterocolitis, periventricular leukomalacia) and the long-term sequelae such as cerebral palsy and chronic lung disease.^1^–^4^

During the course of MIAC, the fetus is bathed in infected amniotic fluid, with evidence of an inflammatory response easily demonstrated by elevated amniotic fluid white blood cell count,^5^ as well as levels of proinflammatory cytokines in fetal plasma and amniotic fluid.^6^–^8^ We have previously designated the complex cascade of clinicopathological events which increase neonatal morbidity and elevate fetal plasma interleukin (IL)-6 over 11 pg/ml as the fetal inflammatory response syndrome (FIRS).^8^ Since amniotic epithelial cells would be in direct contact with infected amniotic fluid, histological chorioamnionitis is a hallmark of a tissue reaction frequently associated with MIAC.^9^

Toll-like receptors (TLRs) are essential components of the innate immune system implicated in recognizing of the presence of microorganisms. So far, 11 TLRs have been identified in mammals and each TLR binds with specific ligands.^9^ TLR-2 predominantly recognizes Gram-positive bacterial components, such as peptidoglycans and the products of Mycoplasma and fungi, while TLR-4 recognizes bacterial endotoxin, which is a component of the cell wall of Gram-negative bacteria.^10^–^12^ A previous study demonstrated the over-expression of both TLR-2 and TLR-4 in the chorioamniotic membranes of patients with infection- and non-infection-related conditions associated with pregnancy,^13^ as is the case with other types of surface epithelia of the body, such as the intestine^14^ and tracheobronchial tree.^15^ Expression of TLR-2 and TLR-4, along with activation of NF-κB, was significantly higher in preterm deliveries with chorioamnionitis,^13^ indicating that engagement of the innate immune system is occurring during normal spontaneous labour as well as during overt inflammatory states.

Human skin, positioned at the interface between the internal milieu and external environment, is endowed with innate immune defence mechanisms against infection.^16^ The epidermis, formed of stratified keratinocytes, is the first line of defence against microbial pathogens. The skin epidermis is not only a mechanical barrier, but also an active immune organ that participates in the protection of the host against microbial invasion by killing pathogenic microorganisms through the production of cationic antimicrobial peptides such as human β-defensins (HBD) and cathelicidin.^17^,^18^ Epidermal keratinocytes also express TLRs, and the expression of TLR-2 and TLR-4 mRNA and proteins has been demonstrated in human keratinocytes along with accessory signalling molecules.^19^,^20^ Microbial killing in the epidermis may be performed by epidermal keratinocytes and by neutrophils attracted to the site of infection by keratinocyte-derived chemokines following TLR activation.^21^

Constant contact with microorganisms in cases of MIAC would explain the significant inflammatory response observed in fetal membranes. While histological chorioamnionitis is a well-defined pathological entity with demonstrated clinical significance on the perinatal outcome,^6^,^22^ little is known about the inflammatory reaction of fetal skin in MIAC. We postulated that MIAC could generate a fetal skin immune response, which is initiated by the recognition of microorganisms by keratinocytes. The purpose of our study was to determine if the fetal skin develops an inflammatory response (dermatitis) and evaluate whether the expression of TLR-2 and TLR-4 is altered in cases of histological chorioamnionitis. This was achieved through the analysis of fetal skin from perinatal autopsy cases.

## Materials and methods

### Tissue samples

Fetal abdominal skin was obtained from fetuses who died shortly after delivery due to immaturity (21–24 weeks' of gestation). All of the cases died within 8 h after birth. The study groups consisted of fetuses born to mothers without (*n* = 4) and with (*n* = 8) histological chorioamnionitis. The diagnosis of histological chorioamnionitis was made as previously described.^8^ All patients' parents provided written informed consent prior to sample collection. The utilization of tissue for research was approved by the Institutional Review Boards of both Wayne State University and the National Institute of Child Health and Human Development.

### Immunohistochemistry

TLR-2, TLR-4, CD15, CD45 and CD68 expression was examined by immunohistochemistry. Fetal skin samples were fixed with 10% buffered formalin for 24 h prior to paraffin embedding for routine histological examination and immunohistochemistry. Serial sections (5 µm thick) were obtained from each case. Deparaffinization, rehydration, antigen retrieval and immunostaining were performed using an automatic immunostainer (Ventana Discovery; Ventana Medical Systems, Inc., Tucson, AZ, USA). The sections were treated with 10% normal horse serum for 20 min. The primary antibodies employed were polyclonal goat anti-TLR-2 and -TLR-4 (1 : 50 and 1 : 60, respectively; Santa Cruz Biotechnology Inc., Santa Cruz, CA, USA) as well as monoclonal mouse anti-CD15 (Dako, Carpinteria, CA, USA), and CD45 (Ventana Medical Systems) and CD68 (Dako). Sections were incubated with biotin-conjugated horse anti-goat (1 : 400; Vector Laboratories, Inc., Burlingame, CA, USA) or biotinylated horse anti-mouse IgG (Universal Vector kit; Vector Laboratories) as a secondary antibody. A diaminobenzidine MAP kit (Ventana Medical Systems) was used for chromogen reaction and counterstaining was performed with Mayer's haematoxylin. Primary antibodies were quenched with excessive amounts of corresponding blocking peptides (Santa Cruz Biotechnology) and used as negative controls. Immunoreactivity for TLR-2, TLR-4, CD15, CD45 and CD68 was evaluated semi-quantitatively and graded as 0 (negative or low numbers of positive cells), 1 (focal scattered immunoreactive cells) and 2 (diffuse and intense infiltration of positive cells) from 5 high-power fields (×400).

### Immunofluorescence

Snap-frozen sections in OCT compound of fetal skin born to mothers without (*n* = 3) and with (*n* = 7) histological chorioamnionitis were used for immunofluorescence for TLR-2, TLR-4, CD3, IL-1α, tumour necrosis factor (TNF)-α, IL-8 and HBDs 1–3. The antibodies used were polyclonal goat anti-TLR-2, anti-TLR-4, anti-HBD-1, anti-HBD-2 and anti-HBD-3 (either 1 : 50 or 1 : 100; Santa Cruz Biotechnology), monoclonal mouse anti-CD3 (1 : 50; Novocastra, Newcastle upon Tyne, UK), anti-IL-1α (1 : 50; eBioscience, San Diego, CA, USA), anti-IL-8 (1 : 50; Abcam Inc., Cambridge, UK) and polyclonal rabbit anti-TNF-α (1 : 50; Santa Cruz Biotechnology). Briefly, the slides were incubated with a serum-free protein blocking solution (Dako) for 20 min at room temperature and incubated with primary antibodies either for 2 h at room temperature or overnight at 4°C. Subsequently, secondary antibodies, FITC-conjugated horse antimouse (Vector Laboratories), donkey anti-goat antibody or donkey anti-rabbit antibody (Biomeda, Foster City, CA, USA) were applied at 1 : 200 dilution for 1 h.

### Statistical analysis

The results are presented as proportions of each grading score according to the study groups and Fisher's exact test was used for comparisons. A *P*-value of <0.05 was considered significant.

## Results

### Microscopic findings

**Figure 1:**
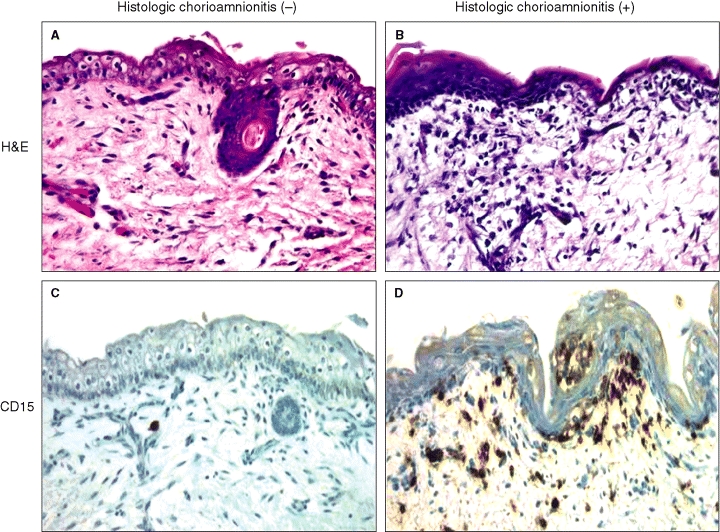
Histological features of abdominal skin obtained from perinatal autopsy cases with and without histological chorioamnionitis. Epidermis and dermis do not show significant changes in a fetus without histological chorioamnionitis (**A,C**). An inflammatory infiltrate is evident in the epidermis and dermis of a fetus with histological chorioamnionitis (**B,D**). The dermoepidermal junction is studded with CD15+ neutrophils.

Clinicopathological characteristics of the cases are summarized in Table 1. All eight cases with histological chorioamnionitis had fetal vasculitis (acute chorionic vasculitis and/or funisitis). Six of these cases had both chorionic vasculitis and funisitis, while two cases had only chorionic vasculitis. Fetal skin samples from the autopsy cases that had histological chorioamnionitis invariably showed perivascular inflammatory cell infiltration in the superficial dermis. The degree of inflammation in fetal skin was less intense than that of the chorioamniotic membranes in individual cases. The inflammatory cells were readily recognized by CD15, CD45 and CD68 immunoreactivity. Dermoepidermal junctions were frequently studded with inflammatory cells and migrating neutrophils were occasionally found in the epidermis (Figure 1A,C). Immunohistochemical analysis showed that the inflammatory infiltrates were composed mainly of CD15+ neutrophils, CD3+ T lymphocytes and CD68+ histiocytes. The fetal skin from pregnancies with histological chorioamnionitis showed greater infiltration of CD15+ and CD68+ cells than those from pregnancies without histological chorioamnionitis (Figure 1B,D; Table 2). A similar observation was made in the mean rate of CD45+ cells, yet the difference did not reach statistical significance (Table 2).

**Table 1:** Clinicopathological profiles of the perinatal autopsies

Case	Sex	Gestational age at delivery (weeks + days)	Birth weight (g)	Clinical diagnosis	Histological chorioamnionitis (CV/F)	AF culture	Survival (h)
1	F	22 + 5	500	pPROM	– (–/–)	ND	1

2	M	22	280	Preeclampsia	– (–/–)	ND	0

3	F	23	500	PTL	– (–/–)	ND	1.5

4	M	22 + 1	410	PTL	– (–/–)	ND	2

5*	M	23 + 2	540	PTL	+ (+/–)	–	7.5

6*	F	21 + 3	400	PTL	+ (+/+)	Ureaplasma	0

7*	M	21 + 6	360	PTL	+ (+/+)	–	3

8*	M	23 + 2	620	PTL	+ (+/–)	–	1.5

9*	F	23 + 4	600	pPROM	+ (+/+)	ND	1.5

10*	M	22	520	pPROM	+ (+/+)	–	0

11*	M	22 + 1	530	PTL	+ (+/+)	Candida	2.5

12*	M	22 + 5	600	pPROM	+ (+/+)	–	1.5

* The cases with fetal dermatitis.

pPROM, Preterm premature rupture of membranes; PTL, preterm labour with intact membranes; CV, chorionic vasculitis; F, funisitis; AF, amniotic fluid; ND, not done.

**Table 2:** Proportions of each grading score of expression of CD15, CD68, CD45, TLR-2, and TLR-4 in the fetal skin

	Fetal skin without chorioamnionitis (*n* = 4)			Fetal skin with chorioamnionitis (*n* = 4)			
			
	Grade 0	Grade 1	Grade 2	Grade 0	Grade 1	Grade 2	*P*-value
CD15	75% (3/4)	25% (1/4)	0% (0/4)	0% (0/8)	62.5% (5/8)	37.5% (3/8)	0.013*

CD68	75% (3/4)	25% (1/4)	0% (0/4)	12.5% (1/8)	25% (2/8)	62.5% (5/8)	0.023*

CD45	0% (0/4)	75% (3/4)	25% (1/4)	0% (0/8)	25% (2/8)	75% (6/8)	> 0.05

TLR-2	75% (3/4)	25% (1/4)	0% (0/4)	0% (0/8)	62.5% (5/8)	37.5% (3/8)	0.013*

TLR-4	0% (0/4)	100% (4/4)	0% (0/4)	0% (0/8)	100% (8/8)	0% (0/8)	> 0.05

* *P* < 0.05, significantly different.

### Expression of TLR-2 and TLR-4

**Figure 2:**
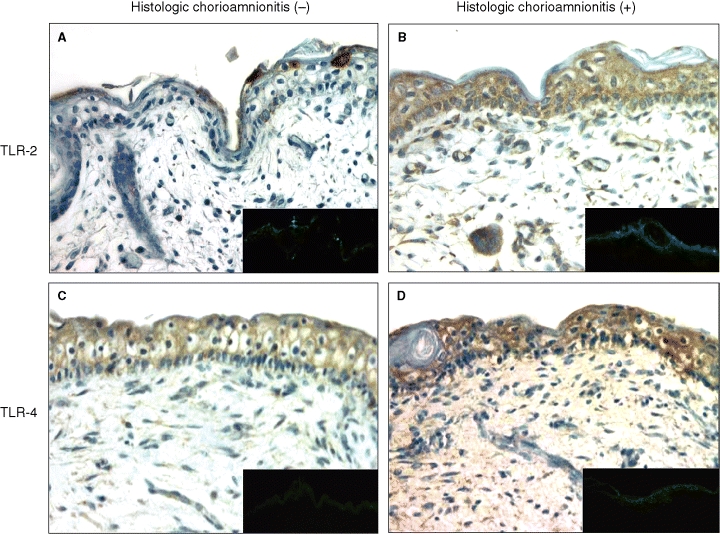
Expression profiles of TLR-2 and TLR-4 in the fetal skin with and without histological chorioamnionitis. In cases without histological chorioamnionitis, faint TLR-2 immunoreactivity (**A**) is found only in the superficial layer of the epidermis, while TLR-4 immunoreactivity (**C**) is found throughout the whole layer of the epidermis. However, both TLR-2 (**B**) and TLR-4 (**D**) are expressed throughout the whole epidermis in cases with histological chorioamnionitis. The increase in TLR-4 immunoreactivity is not as prominent as that of TLR-2. Insets show immunofluorescence staining results with corresponding antibodies.

**Figure 3:**
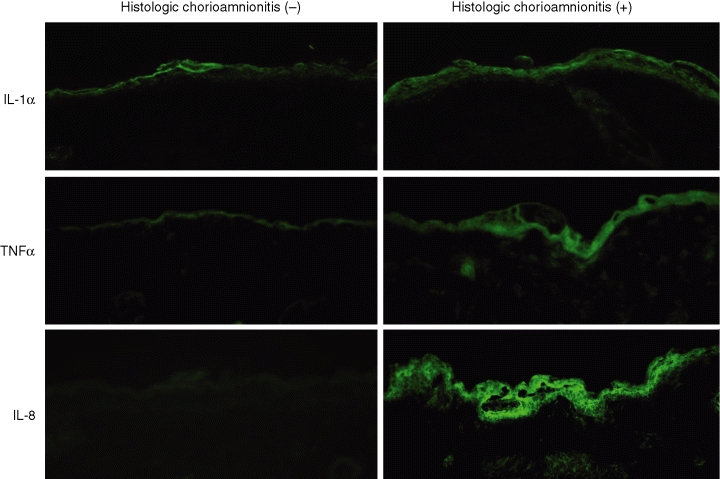
Expression of proinflammatory cytokines [interleukin (IL)-1α, tumour necrosis factor (TNF)-α] and chemokine (IL-8) in the fetal skin. Expression of IL-1α, TNF-α and IL-8 is markedly increased in the fetal epidermis of a case with histological chorioamnionitis, compared with that of a case without this condition.

**Figure 4:**
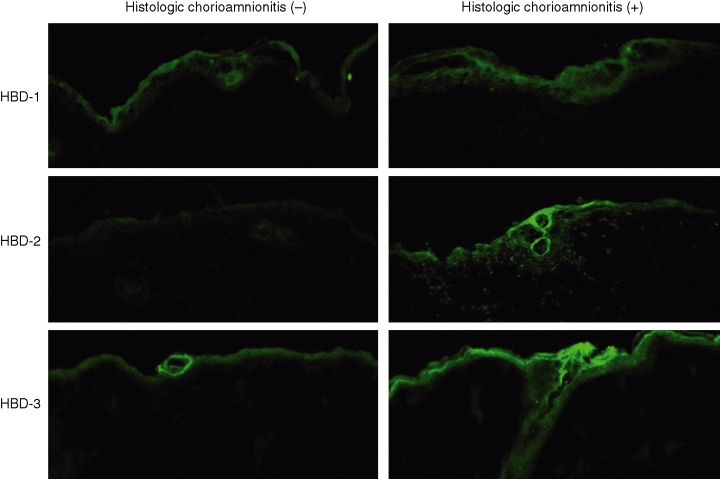
Expression of antimicrobial peptide human β-defensins (HBD)-1, HBD-2 and HBD-3 in fetal skin. Increases in HBD-2 and HBD-3 expression are more prominent in a case affected with histological chorioamnionitis, compared with a case without this condition. HBD-2 and HBD-3 expression seems to be more inducible in nature.

The fetal skin expressed TLR-2 and TLR-4 in the epidermis regardless of histological chorioamnionitis status. However, the expression of TLR-2 was significantly higher in the epidermis of fetuses with histological chorioamnionitis than in those without this condition (Figure 2A,B; Table 2). Significant TLR-2 immunoreactivity was found only in the keratinocytes in the superficial layer of the epidermis in cases without histological chorioamnionitis (Figure 2A). In contrast, the majority of the epidermal keratinocytes in the basal through to the superficial layers were strongly immunopositive for TLR-2 in fetuses with histological chorioamnionitis (Figure 2B). TLR-2 immunoreactivity was also readily detected in dermal capillary endothelial cells and hair follicles. TLR-4 immunoreactivity in the keratinocytes was constitutive and diffuse throughout the whole layer of epidermis regardless of whether or not histological chorioamnionitis was present (Figure 2C,D; Table 2). Cases with histological chorioamnionitis also showed a marked increase in the expression of proinflammatory cytokines (IL-1α, TNF-α) and chemokine IL-8 (Figure 3). Increases in IL-8 immunofluorescence were especially prominent and were observed throughout the whole thickness of the epidermis. Among the HBDs, HBD-1 immunoreactivity was observed throughout the entire epidermis of cases both with and without histological chorioamnionitis. In contrast, HBD-2 and HBD-3 were expressed only by keratinocytes in the superficial epidermis in cases without histological chorioamnionitis, while distinct immunoreactivity was found throughout the epidermis in the cases with histological chorioamnionitis (Figure 4).

## Discussion

We have shown that fetal dermatitis is present in cases of histological chorioamnionitis. This *in utero* process observed in mid-trimester pregnancies is associated with increased expression of TLR-2 and TLR-4 and concomitant up-regulation of cytokines/chemokines and antimicrobial factors in epidermal keratinocytes. Our observations suggest that fetal keratinocytes are actively involved in the fetal inflammatory response and demonstrate that a unique pathological entity (fetal dermatitis) can occur in histological chorioamnionitis.

Funisitis and chorionic vasculitis are the histological counterparts of FIRS in that these processes represent fetal vasculitis and show a strong correlation with fetal plasma IL-6 levels.^8^ However, in terms of anatomical considerations, these processes represent extrafetal inflammation. We believe that the fetal skin represents a primary physiological barrier capable of responding to the presence of microbial factors and protecting against MIAC. Our findings further support our hypothesis that this process is of the utmost significance as a response to MIAC, since it represents a purely intrafetal inflammatory response (although the number of cases in the present study was not sufficient to assess its relationship with FIRS).

The gold standard for the diagnosis of MIAC is a positive amniotic fluid culture for bacteria or genital Mycoplasmas.^23^ Microorganisms (Candida species and *Ureaplasma urealyticum*) were demonstrated in only 2 out of 7 cases with chorioamnionitis in which amniotic fluid cultures were performed, making it difficult to define cases complicated by MIAC through cultures alone. However, the presence of chorionic vasculitis and/or funisitis represents evidence for the existence of MIAC. It is noteworthy that standard clinical microbiological culture has limitations and is not sensitive enough to detect all cases of MIAC. For example, amniotic fluid cultures missed 40% of patients with microbial invasion by *U. urealyticum* as determined by polymerase chain reaction in cases with preterm premature rupture of membranes.^24^

Expression of TLR-2 and TLR-4 was dependent upon the differentiation status of human keratinocyte cell line (HaCaT). It seems logical that there should be a gradient of TLRs in the epidermis since pathogens are present in the upper, more differentiated layers.^25^ We found a gradient of TLR-2 in fetal epidermis that was not exposed to MIAC. In cases with histological chorioamnionitis, however, significant TLR-2 expression was found in all the epidermal layers. Lipopolysaccharide and interferon-γ treatment of human keratinocytes *in vitro* increases the expression of TLR-2 50-fold, and TLR-4 5-fold.^21^ The relatively marked up-regulation of TLR-2 in the fetal epidermis compared with TLR-4 in our study is consistent with this *in vitro* observation. Expression patterns of TLR-2 seem to be more inducible, while that of TLR-4 in epidermis is more constitutive in human keratinocytes. Although their expression patterns seem to differ slightly, there is emerging evidence of cross-talk between TLR-4 and TLR-2. TLR-4 activation by lipopolysaccharide up-regulates the expression of TLR-2 in endothelial cells.^26^ The changes in TLR-2 and TLR-4 expression are basically identical to those of the fetal membranes affected with histological chorioamnionitis.^13^ Functionally competent activation of the TLR signalling pathway is shown through associated changes in proinflammatory cytokines and the chemokine IL-8.

The histopathological intensity of dermatitis in fetal skin was much less severe compared with that of chorioamnionitis in the chorioamniotic membranes. This may be related to the relative immunological immaturity of the fetus. Even in placental inflammation, fetal membranitis, whose inflammatory infiltrates are of maternal origin, almost inevitably precedes chorionic vasculitis or funisitis, which represents fetal vasculitis.^27^ Characterization of the immunophenotype of the inflammatory cells revealed mixed-type infiltrates composed of neutrophils, lymphocytes and histiocytes, suggesting chronicity of the inflammatory process. Among the generalized activation of immune mechanisms, prominent up-regulation of IL-8 by keratinocytes is noteworthy.^21^ This finding strongly implies that fetal epidermal keratinocytes contribute in a significant way to the increase of IL-8 in amniotic fluid, which then enhances neutrophilic migration into the amniotic cavity.^21^,^28^ The neutrophils in intra-amniotic inflammation are fetal in origin.^29^ The presence of migrating neutrophils in fetal epidermis, along with strong IL-8 expression by keratinocytes, suggests that the fetal skin can also be a significant transmigratory route for fetal neutrophils, along with chorionic plate vessels and the umbilical cord, which we have proposed as potential sites of origin of amniotic fluid neutrophils.^30^ In this context, cases with increased amniotic fluid white blood cell counts in the absence of histological chorioamnionitis may be potential candidates for transmigration of fetal leucocytes across the skin.

In summary, we report for the first time that fetal dermatitis is a distinct and unique pathological entity associated with MIAC, and probably represents an early stage of FIRS. We define the condition as ‘FIRS-associated fetal dermatitis’, as it is the fetal counterpart of placental histological chorioamnionitis and shares a common pathogenesis. The study also clearly shows that fetal skin, even in the mid trimester, is capable of recognizing microbial products through pattern recognition receptors TLR-2 and TLR-4 and, thus may actively participate in innate immune defence. Recent reports on early postnatal skin colour changes in term newborns with subclinical histological chorioamnionitis also provide interesting evidence that biological alterations in fetal skin are occurring in association with chorioamnionitis.^31^ Finally, the findings of our study also emphasize the significance of meticulous examination of the fetal skin during routine perinatal autopsies.

## Abbreviations

FIRS: fetal inflammatory response syndrome
HBD: human β-defensins
IL: interleukin
MIAC: microbial invasion of the amniotic cavity
TLR: Toll-like receptor
TNF: tumour necrosis factor
